# Assessing pH‐dependent activities of virulence factors secreted by *Candida albicans*

**DOI:** 10.1002/mbo3.1342

**Published:** 2023-01-03

**Authors:** Asier Ramos‐Pardo, Rocío Castro‐Álvarez, Guillermo Quindós, Elena Eraso, Elena Sevillano, Vladimir R. Kaberdin

**Affiliations:** ^1^ Department of Immunology, Microbiology and Parasitology University of the Basque Country UPV/EHU Leioa Spain; ^2^ IKERBASQUE, Basque Foundation for Science Bilbao Spain; ^3^ Research Centre for Experimental Marine Biology and Biotechnology (PIE‐UPV/EHU) Plentzia Spain

**Keywords:** *Candida* pathogenicity, clinical isolates, hydrolytic zones, secreted enzymes

## Abstract

*Candida albicans* is an opportunistic pathogen that can thrive under adverse conditions including suboptimal pH, nutrient scarcity, and low levels of oxygen. Its pathogenicity is associated with the production of virulence factors such as extracellular hydrolytic enzymes and toxins. This study was aimed at determining the effect of external pH, substrate nature, and strain origin on protease, lipase, and hemolysin production. To achieve this objective, agar plate assays were performed at pH 5.0, 6.5, and 7.5 with substrates suitable for the detection of each family of enzymes. Moreover, the study was conducted with 20 clinical *C. albicans* isolates from blood, oral cavity, skin, urine, and vagina. The hydrolytic zones formed around the colonies were further measured to calculate the *Ez* (enzymatic zone) indexes. We found that detection of proteases in skim milk agar plates was possible for most isolates only at pH 5 (80%) and pH 6.5 (75%), whereas BSA plates could confer protease detection exclusively at pH 5 (80%). Similarly, the percentage of isolates possessing lipolytic activities was higher at pH 5 (90%) than at pH 6.5 (70%) and pH 7.5 (35%). In contrast, hemolytic activities were detected in all isolates at pH 6.5 and 7.5 but not at pH 5. Further analysis revealed that some differences in the detected activities could potentially be attributed to the anatomical origin of these isolates. Collectively, these findings suggest that the pH of the site of infection might be critical for mimicking the microenvironment employed to experimentally discover the key virulence factors.

## Graphical abstract

The pathogenicity of Candida albicans is associated with the production of virulence factors such as extracellular hydrolytic enzymes and toxins. The results reported here demonstrate that the activity of secreted hydrolytic enzymes, in particular proteases, lipases, and hemolysins, is generally dependent on pH and is potentially associated with the clinical origin of *C. albicans* isolates.

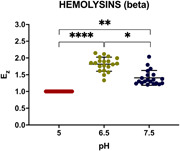

## 1 INTRODUCTION

The *Candida* genus is widely present in mammals' mycobiome. Although many *Candida* species belong to the common skin and mucosa mycobiomes (Harpf et al., 2020), their interaction with the host can occasionally trigger the infectious disease known as candidiasis. This disease often occurs in immunocompromised persons or those suffering from dysbiosis (Ortega‐Riveros et al., 2017; Raesi Vanani et al., 2019), especially, when the natural resistance to infections and/or physiological conditions of the host cannot neutralize the action of fungal virulence factors, thus making it possible for the commensal *Candida* to act as an opportunistic pathogen (Marcos‐Arias et al., 2011).

Although there are more than 150 *Candida* species, candidiasis is most frequently caused by *Candida albicans*, *Candida glabrata*, *Candida tropicalis*, *Candida parapsilosis*, or *Candida krusei* (Henriques & Williams, 2020; Quindós et al., 2018; Turner & Butler, 2014). These pathogenic *Candida* species often elicit noninvasive candidiasis typically localized in the oropharyngeal cavity, skin, vaginal, and gastrointestinal tracts. In addition, these fungi can also enter the bloodstream through intravenous catheters or by infiltration/translocation through the intestinal mucosal barrier, thus leading to candidemia and other forms of invasive candidiasis caused by the dissemination of *Candida* in different tissues and organs. Invasive candidiasis is particularly severe, as it is usually associated with an increase in morbidity and mortality.

*Candida* species employ a large arsenal of virulence factors to elicit infections (Whittington et al., 2014). Their specific role in disease can vary and depends on the site of infection and the morphological state of the fungus. Different *C. albicans* morphologies, such as blastospore, hyphae, or pseudohyphae, can greatly influence infection and disease development by (i) facilitating cell adhesion, penetration/translocation through the intestinal mucosal barrier, and dissemination into the bloodstream during early stages of infection or (ii) by suppressing host immune responses (Chow et al., 2021).

Several molecular virulence factors including adhesins, invasins, and hydrolytic enzymes play essential roles in candidiasis. The major hydrolytic enzymes include proteases, lipases, and hemolysins (Mayer et al., 2013). Among different proteolytic enzymes, aspartic proteases (E.C. 3.4.23) (Kashparov et al., 1998) belong to the best‐studied ones in *Candida*. The key feature of these bilobed globular enzymes is the presence of two aspartic residues and a water molecule in their catalytic site (Mandujano‐González et al., 2016). Aspartic proteases in *Candida* form two families: (i) secreted enzymes that are also known as candidapepsines (SAP), and (ii) surface‐exposed ones (i.e., yapsins), which remain attached to the cell wall. In *C. albicans*, 10 *SAP* genes (*SAP1* to *SAP10*) have been described so far, and the corresponding enzymes have been further grouped according to the pH range, at which they manifest their optimal activities (Aoki et al., 2011). The optimal pH for the first group of enzymes (i.e., Sap2, Sap3, and Sap8) is 2.5‐4, whereas the second one (i.e., Sap1, Sap4, Sap5, Sap6, Sap7, Sap9, and Sap10) is most active at pH 5–6.5. Secreted *Candida* proteases exert a number of biological functions including their participation in the initial steps of colonization by damaging host tissues and digestion of the host proteins to generate nutrients for *Candida.* In addition, proteases can help the pathogen to escape from the host immune system by degrading immunoglobulin A, antimicrobial peptides, or inducing oxidative burst in the host immune system cells, thereby inducing apoptosis (Rapala‐Kozik et al., 2018). Although some SAPs have also been reported (Forsberg et al., 2019; Rapala‐Kozik et al., 2018) in other *Candida* species (i.e., *C. tropicalis*, *C. parapsilosis*, *C. dubliniensis*, and *Candida auris*), little is known about their biochemical characteristics and biological functions. The family of yapsins, which is less characterized than candidapepsines, is widely present in *C. glabrata*, a species that lacks secreted aspartic proteases. The cell‐wall‐attached yapsins contribute to cell wall formation and play a significant role in *Candida* adhesion and biofilm formation (Rapala‐Kozik et al., 2018).

Another family of virulence factors includes extracellular lipases (EC. 3.1.1.3). Lipase activity in *C. albicans* was first reported by Werner in 1965, and since then, different studies have revealed at least ten different lipase isoforms of 20–60 kDa encoded by *LIP* genes (*LIP1–LIP10*, respectively) (Hube et al., 2000). *Candida albicans* lipases share common structural elements that include an α/β‐hydrolase fold, a catalytic triad composed of Ser‐His‐Asp/Glu, and a cap structure. *Candida* lipases are stable over a wide pH range (4–11) (Mehta et al., 2017) with optimal activity at pH 7–9 (González‐Bacerio et al., 2010). Nevertheless, some lipases are primarily active at more acidic or alkaline pH. The optimal temperature, at which they show greater activity, is between 35°C and 50°C, even though certain thermostable lipases can efficiently process their substrates at temperatures higher than 70°C (González‐Bacerio et al., 2010). Similar to proteases, lipases play a key role in *Candida* virulence contributing to adhesion and tissue damage, and negatively affecting immune response, delaying inflammatory cascade, and thus facilitating *Candida* invasion and survival in host tissues (Ciurea et al., 2020).

In addition to the production of proteases and lipases, *Candida* also secrets various hemolysins, initially identified in *C. albicans* by Manns et al. (1994) and subsequently found in other species such as *C. tropicalis* (Favero et al., 2011) and *C. parapsilosis* (Favero et al., 2014). Hemolysins are pore‐forming toxins that recognize specific ligands on the surface of the target cells (Gonzalez et al., 2008). These virulence factors help *Candida* to damage the host cells rich in iron, in particular erythrocytes, thus providing access to the host proteins with a high concentration of iron (e.g., hemoglobins) during candidiasis (Noble, 2013). The use of agar plates supplemented with blood to monitor hemolysin activities made it possible to discern two major types of hemolysin activities. The first, α‐hemolysis, which is associated with incomplete lysis of blood cells due to partial damage of the erythrocyte membrane, visually produces various subtypes of greenish (or brownish/opaque) halos around the fungal colony (Savardi et al., 2018), whereas the second, β‐hemolysis, represents the complete lysis of erythrocytes revealed by clear translucent halos (Hogg, 2013).

The broad spectrum and location of infections that are caused by *Candida* spp. indicate that these pathogens can thrive in very diverse environments (i.e., at different pH, temperature, access to nutrients, atmospheric oxygen, or exposure to the immune system) faced by the microorganisms in the sites of infection. Although some environmental factors (e.g., pH and oxygen availability) can alter the potential of *Candida* to elicit infections, the actual impact of these parameters is still poorly characterized. The main goal of this study was to investigate the impact of pH on the activity of secreted virulence factors (viz., proteases, lipases, and hemolysins) known for their role in *Candida*‐associated diseases and assess if their activity is influenced by the strain origin.

## 2 MATERIALS AND METHODS

### 2.1 Culturing and storage of *Candida* isolates and control strains

#### 2.1.1 Candida isolates and control strains

In the course of this study, 20 clinical isolates of *C. albicans* from five different infection sites (oral cavity, urine, vaginal tract, skin, and blood) as well as control strains were analyzed (Table 1). The strains selected as positive controls for protease plate assays were *C. albicans* NCPF 3153 and *C. tropicalis* NCPF 3111, whereas *C. parapsilosis* ATCC 22019 and *C. albicans* ATCC 90028 were used as positive controls for lipase and hemolysin assays, respectively. The control strains belong to the American Type Culture Collection (ATCC) and National Collection of Pathogenic Fungi (NCPF) of the United States and the United Kingdom, respectively.

**Table 1:** *Candida* isolates and control strains used in this study

	Species	Source					
		Oral cavity	Urine	Vaginal area	Skin	Blood	Control
Isolate/Strain ID	*Candida albicans*	08‐052	13‐008	18‐100	05‐126	19‐002	NCPF 3153
		19‐078	15‐153	18‐105	93‐432	18‐012	ATCC 90028
		19‐010	07‐154	18‐135	16‐136	18‐028	
		18‐034	13‐028	18‐137	15‐159	18‐020	
	*Candida parapsilosis*						ATCC 22019
	*Candida tropicalis*						NCPF 3111

#### 2.1.2 Candida culturing and storage

All experiments were performed with fresh inocula prepared from colonies cultured on Sabouraud dextrose agar (SDA; Scharlab S.L.). The latter were obtained by inoculation of plates with aliquots (ca. 30 μL) of *Candida* stocks followed by incubation at 37^o^C for 24–48 h. The cells from several colonies formed on SDA were suspended in a sterile storage solution containing 0.6% yeast extract (Conda Pronadisa), 1.2% peptone (Condalab), 1.2% dextrose (LabKem), and 40% v/v glycerol and were either stored at −80^o^C in cryovials or kept refrigerated at −4^o^C for daily use.

### 2.2 Plate assays for detection of secreted hydrolytic and hemolysin‐like activities

Plates for the detection of secreted enzymes were prepared as described in Section 2.2.1, inoculated with control and test strains, and incubated to observe hydrolysis zones (halos) around the colonies as detailed in Section 2.2.2. The activities of the secreted enzymes were semiquantified (see Section 2.2.3) by calculating an enzymatic zone coefficient (*Ez*) based on the ratio of the diameter of the halo to the diameter of its cognate colony. The experiments were done in triplicate to ensure reproducibility and minimize inter‐plate variability.

#### 2.2.1 Preparation of plates

Detection of hydrolytic and hemolysin‐like activities included the preparation of plates with different media specifically formulated to detect these enzymes at different pH. Before autoclaving, the pH of each medium was adjusted to pH 5, 6.5, and 7.5 using 1 M HCl or 1 M NaOH. Then, the pH values were verified after autoclaving and corrected (i.e., re‐adjusted), if necessary.

To detect protease activity, two media were prepared based on the protocol developed by Cassone et al. (1987, 1995). Both media contained 2% European Bacteriological agar (Condalab), 1.17% yeast carbon base (Sigma‐Aldrich), 0.01% yeast extract and were further supplemented either with 0.2% skim milk (VWR) or 0.2% BSA (Sigma‐Aldrich), respectively. In contrast to the addition of skim milk before autoclaving, BSA was added as a 20% sterile BSA stock solution to the second medium only after its autoclaving and cooling to 50^o^C.

Lipase plate assays were performed employing a slightly modified medium developed by Slifkin (2000). Following this protocol, 4.5% malt agar (Sigma‐Aldrich), 5.84% NaCl, 0.056% CaCl_2_, and 2% Tween® 80 (Sigma‐Aldrich) were dissolved in distilled water and autoclaved before casting the plates.

*Candida* hemolysin production was assessed using the plate assay described by Manns et al. (1994) and Luo et al. (2001) with some modifications. Namely, this medium contained 6.5% SDA, 3% dextrose, and 7% defibrinated sheep blood (Thermo Scientific). The first two components (SDA and dextrose) were dissolved in distilled water and pH was adjusted before autoclaving. The autoclaved medium was cooled down to 50^o^C and blood was added.

#### 2.2.2 Inoculation and incubation of plates

Before inoculation, cell suspensions with turbidity 0.8 McFarland units (i.e., approximately 10^7^ cells/mL) were prepared by suspending *Candida* cells in a 0.85% NaCl saline solution (bioMérieux S.A.). Plates were inoculated by placing 10 μL of each cell suspension and then incubated at 37^o^C for 5 days (skim milk‐containing plates), 7 days (BSA‐containing plates), or 6 days (Tween 80‐containing plates) under aerobic conditions, whereas hemolysin detection was carried out at 37^o^C for 2 days in an anaerobic jar containing CO_2_ Gen™ 2.5 L Sachet (Thermo Scientific) to create a 5% CO_2_‐rich atmosphere.

#### 2.2.3 Interpretation of activity halos

Measurements and comparisons of secreted enzymatic activities were carried out according to the procedure described by Price et al. (1982). Briefly, the diameters of halos and colonies were measured and further used in this equation to calculate the *Ez* index:

Ez=Diameter of hydrolysis zone÷Diameter of the colony

### 2.3 Statistical analysis

The data obtained were statistically analyzed as described previously (Motulsky, 2016) and presented as graphs by using GraphPad Prism 7.0 (GraphPad Software). Briefly, an analysis of the supposed normal distribution of the data was carried out by performing a D'Agostino‐Friedman test. In addition, post hoc Dunn's multiple comparisons tests were performed to establish if statistically significant differences in the tested activities were observed at different pH. Both tests were also employed to assess whether the putative associations between the pH‐dependent enzymatic activities and strain origin were statistically significant. After setting the threshold (*ɑ*) at 0.05, data with a *p*‐value lower than or equal to *ɑ* were considered to be statistically significant.

## 3 RESULTS

To assess the effect of pH on the activity of secreted virulence factors, 20 *C. albicans* clinical isolates (see Table 1) from various infection sites (i.e., blood, oral cavity, skin, urine, and vaginal tract) that differ in their physiological pH as well as four control strains (two *C. albicans*, and one each *C. tropicalis* and *C. glabrata*) were used in this study. The activity of three major secreted virulence factors (proteases [see Section 3.1], lipases [see Section 3.2], and hemolysins [see Section 3.3]) was evaluated by using plate assays designated to separately detect each type of activity.

### 3.1 Detection of secreted proteases

#### 3.1.1 Detection of secreted proteases at different pH using control strains

Before carrying out plate assays with clinical isolates, we tested the efficiency of two different solid media (BSA‐ and skim milk‐containing agar) in the detection of proteases secreted by the control strains at pH 5.0, 6.5, and 7.5 under aerobic conditions. These experiments were done with 4 control strains (see Table 1) using skim milk and BSA agar plates incubated at 37°C for 5 and 7 days, respectively. We found that only *C. tropicalis* NCPF 3111 on skim milk agar plates was able to secret proteases detectable at all three pH. In contrast, secretion of proteases by *C. albicans* NCPF 3153 was detected only at pH 6.5 and 7.5, while *C. albicans* ATCC 90028 yielded a proteolytic zone (halo) only at pH 5. As for BSA‐containing agar plates, *C. tropicalis* NCPF 3111, *C. albicans* NCPF 3153, and *C. albicans* ATCC 90028 produced halos only at pH 5, while the proteolytic activity of *C. parapsilosis* ATCC 22019 was not detected at any tested pH. Moreover, skim milk was the only substrate suitable for the detection of secreted proteases at pH 6.5. Interestingly, there was a clear difference in the appearance of halos formed on these two different media. While the halos formed on skim milk agar plates were transparent, those on BSA‐containing plates were white and opaque.

#### 3.1.2 Detection of proteases secreted by *C. albicans* clinical isolates at different pH

**Figure 1:**
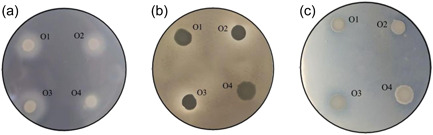
Detection of proteases secreted by oral *Candida albicans* isolates 08‐052 (O1), 18‐034 (O2), 19‐078 (O3), and 19‐010 (O4). Assays were carried out using BSA‐containing agar plates at pH 5 (a) and skim milk‐containing agar plates at pH 6.5 (b) and pH 5 (c).

Once the protocol for the detection of proteolytic activities secreted by the control strains was tested, we used it for assaying a representative group of *C. albicans* isolates from various sites of infection (Table 1). The images obtained with the oral isolates are presented in Figure 1 **,** whereas the remaining data are summarized in Table 2. As seen in this table, the assay carried out with skim milk agar plates revealed that 16 (80%) and 15 (75%) isolates showed protease activity at pH 5 and 6.5, respectively. In contrast, no protease activity was observed at pH 7.5. Moreover, analysis of these data did not reveal any significant association between the origin of the clinical isolates tested and their capacity to produce proteolytic halos. Regarding the assays on BSA plates, protease activities were detected only at pH 5.

**Table 2:** Detection of secreted proteases of clinical *Candida albicans* isolates from blood, oral cavity, skin, urine, and vagina using microbiological media containing skim milk or BSA

	Substrate	Skim milk			BSA				Substrate	Skim milk			BSA		
pH		5.0	6.5	7.5	5.0	6.5	7.5	pH		5.0	6.5	7.5	5.0	6.5	7.5
		Blood								Oral cavity					
Isolate ID	18‐012	+	+	‐	+	‐	‐	Isolate ID	08‐052	‐	+	‐	+	‐	‐
	18‐020	+	+	‐	+	‐	‐		18‐038	+	+	‐	+	‐	‐
	18‐028	+	‐	‐	+	‐	‐		19‐010	‐	+	‐	+	‐	‐
	19‐002	+	+	‐	+	‐	‐		19‐078	+	+	‐	+	‐	‐
		Skin								Urine					
Isolate ID	05‐126	+	+	‐	+	‐	‐	Isolate ID	07‐154	+	+	‐	+	‐	‐
	15‐159	+	+	‐	‐	‐	‐		13‐008	+	+	‐	+	‐	‐
	16‐136	+	+	‐	‐	‐	‐		13‐028	‐	‐	‐	+	‐	‐
	93‐432	+	+	‐	+	‐	‐		15‐153	‐	‐	‐	‐	‐	‐
		Vagina													
Isolate ID	18‐137	+	‐	‐	+	‐	‐								
	18‐100	+	+	‐	+	‐	‐								
	18‐105	+	‐	‐	‐	‐	‐								
	18‐135	+	+	‐	+	‐	‐								

*Note*: The presence and absence of detectable protease activities are indicated by “+” and “‐,” respectively.

**Figure 2:**
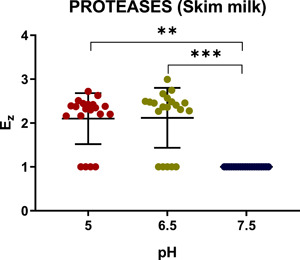
Dependence of the enzymatic protease activity coefficient (*Ez*) of *Candida albicans* isolates on the tested pH of milk agar. Scattered red (pH 5), green (pH 6.5), and blue (pH 7.5) circles represent the studied strains. Horizontal bars represent the statistically reliable (*ɑ* = 0.05) mean (longer bar) and standard deviation (shorter bars) values. Brackets indicate statistically reliable sets of data characterized by two thresholds: **p ≤ 0.01 and ***p ≤ 0.001.

**Figure 3:**
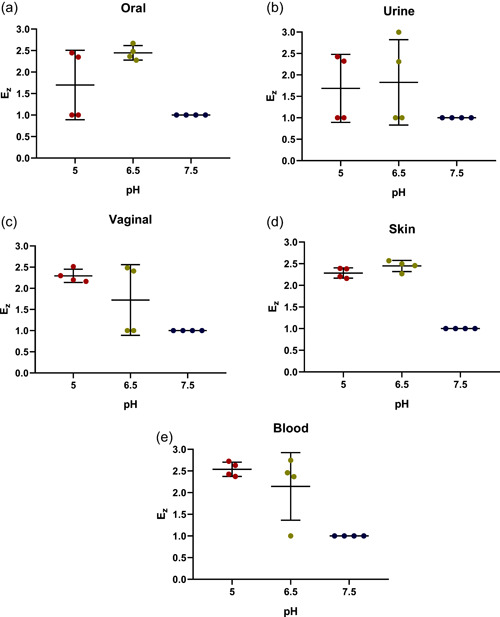
Dependence of the protease activity coefficient (*Ez*) of *Candida albicans* isolates on pH. Assays with *Candida albicans* isolates from the oral cavity (a), urine (b), vagina (c), skin (d), and blood (e) were carried out on milk agar plates at pH 5, 6.5, and 7.5. Scattered red (pH 5), green (pH 6.5), and blue (pH 7.5) circles represent the studied strains. Horizontal bars represent the mean (longer bars) and standard deviation (shorter bars) values.

Comparison of the enzymatic indexes corresponding to protease activity of *C. albicans* isolates tested on skim milk agar showed statistically supported (*p* < 0.0001) differences in the proteolytic activity detected at pH 7.5 and those at pH 5 and 6.5 (Figure 2). As to protease activity detected on BSA‐containing plates (Table 2), it was observed only at pH 5, thus making it impossible to compare proteolytic activities at different pH. According to the results of the corrected Dunn's multiple comparisons test, we did not find any statistically significant dependence of protease activity on the site of infection at any of the three pH values using either skim milk or BSA agar plates (Figure 3).

### 3.2 Detection of secreted lipases

#### 3.2.1 Testing the pH‐dependent activity of lipases secreted by control strains

Before using malt agar with pH 5, 6.5, and 7.5 at 37°C for detection of lipase activity in clinical isolates, the media were initially tested using control strains. The assays were performed at 37^o^C by incubating plates for 6 days under aerobic conditions. Except for *C. tropicalis* NCPF 3111, which showed no lipase activity at pH 5 and 7.5, the rest of the control strains yielded positive results at every pH.

#### 3.2.2 Detection of lipases secreted by *C. albicans* clinical isolates at different pH

**Figure 4:**
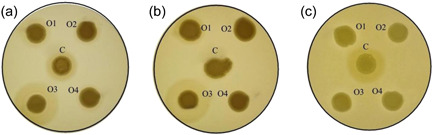
Detection of lipases secreted by oral *Candida albicans* isolates 08‐052 (O1), 18‐034 (O2), 19‐010 (O3), 19‐078 (O4), and *C. parapsilosis* (ATCC 22019) (C). The assay was carried out at pH 5 (a), pH 6.5 (b), and pH 7.5 (c).

**Figure 5:**
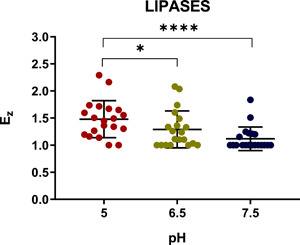
Dependence of the enzymatic activity coefficient (*Ez*) of *Candida albicans* isolates on the tested pH. Scattered red (pH 5), green (pH 6.5), and blue (pH 7.5) circles represent the studied strains. Horizontal bars represent the statistically reliable (*ɑ* = 0.05) mean (longer bar) and standard deviation (shorter bars) values. Brackets indicate statistically distinct sets of data with two thresholds (*p ≤ 0.05 and ****p ≤ 0.0001).

**Figure 6:**
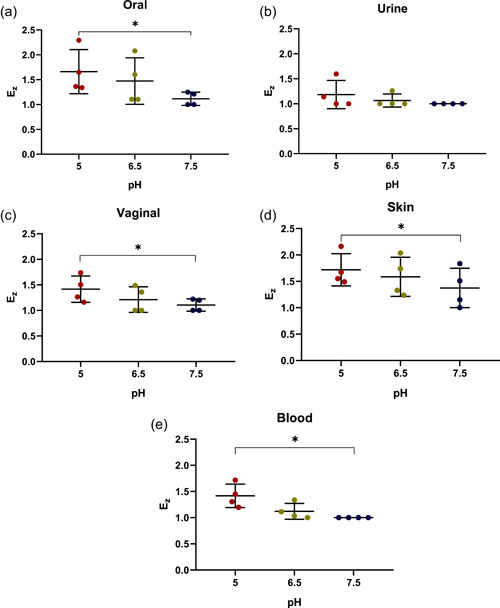
Dependence of the lipase activity coefficient (*Ez*) of *Candida albicans* isolates on pH. Assays with *Candida albicans* isolates from the oral cavity (a), urine (b), vagina (c), skin (d), and blood (e) were carried out on malt agar plates at pH 5, 6.5, and 7.5. Scattered red (pH 5), green (pH 6.5), and blue (pH 7.5) circles correspond to the studied isolates. Horizontal bars represent the statistically reliable (*ɑ* = 0.05) mean (longer bar) and standard deviation (shorter bars) values. Brackets indicate statistically distinct sets of data (*p ≤ 0.05).

Following the assays with the control strains, we next tested a representative collection of *C. albicans* strains for lipase activity secreted at different pH. The images obtained with the strains isolated from the oral cavity are shown in Figure 4, whereas the remaining data are summarized in Table 3. As seen in this table, 18 (90%) and 14 isolates (70%) possessed lipase activity at pH 5 and 6.5, respectively. In contrast, only seven isolates showed lipase activity at pH 7.5. Moreover, there was no evidence that the lack of detectable lipase activity was associated with the clinical origin of the lipase‐negative isolates. The subsequent comparison of lipase activities determined for all *C. albicans* clinical isolates at different pH revealed statistically significant (*p* < 0.0001) differences in the mean value of *Ez* (Figure 5). However, further analysis of these data applying the corrected Dunn's test did not provide any strong support for a dependence of these mean values on the origins of isolates (Figure 6).

**Table 3:** Detection of secreted lipases of clinical *Candida albicans* isolates from blood, oral cavity, skin, urine, and vagina using malt agar supplemented with Tween 80

	Substrate	Tween 80				Substrate	Tween 80		
pH		5.0	6.5	7.5	pH		5.0	6.5	7.5
	Blood					Oral cavity			
Isolate ID	18‐012	+	+	‐	Isolate ID	08‐052	+	+	+
	18‐020	+	‐	‐		18‐038	+	+	‐
	18‐028	+	+	‐		19‐010	+	+	+
	19‐002	+	+	‐		19‐078	+	+	‐
	Skin					Urine			
Isolate ID	05‐126	+	+	‐	Isolate ID	07‐154	‐	‐	‐
	15‐159	+	+	+		13‐008	+	‐	‐
	16‐136	+	+	+		13‐028	+	+	‐
	93‐432	+	+	+		15‐153	‐	‐	‐
	Vagina								
Isolate ID	18‐137	+	+	+					
	18‐100	+	+	+					
	18‐105	+	‐	‐					
	18‐135	+	‐	‐					

*Note*: The presence and absence of detectable lipase activities are indicated by “+” and “‐,” respectively.

### 3.3 Detection of secreted hemolysins

#### 3.3.1 Testing the pH‐dependent activity of hemolysins secreted by control strains

Before carrying out assays with clinical isolates, experiments were done to set up the protocol using the control strains, which were incubated for two days on blood agar plates with pH 5, 6.5, and 7.5 at 37°C in the presence of 5% CO_2_. We found that *C. tropicalis* NCPF 3111, *C. albicans* NCPF 3153, and *C. albicans* ATCC 90028 produced hemolysis zones at pH 6.5 and 7.5. Unlike the above control strains, *C. parapsilosis* ATCC 22019 had difficulties growing on blood agar plates and did not show any detectable hemolysin activity at all.

#### 3.3.2 Detection of hemolysins secreted by *C. albicans* isolates at different pH

**Figure 7:**
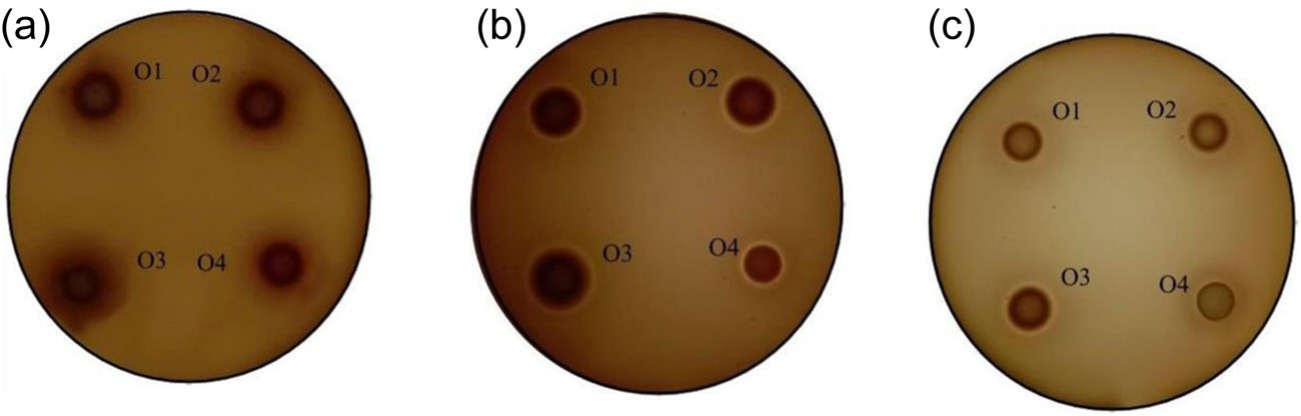
Detection of hemolysins secreted by oral *Candida albicans* isolates 08‐052 (O1), 18‐034 (O2), 19‐078 (O3), and 19‐010 (O4). Assays were carried out at pH 5.0 (a), pH 6.5 (b), and pH 7.5 (c).

**Figure 8:**
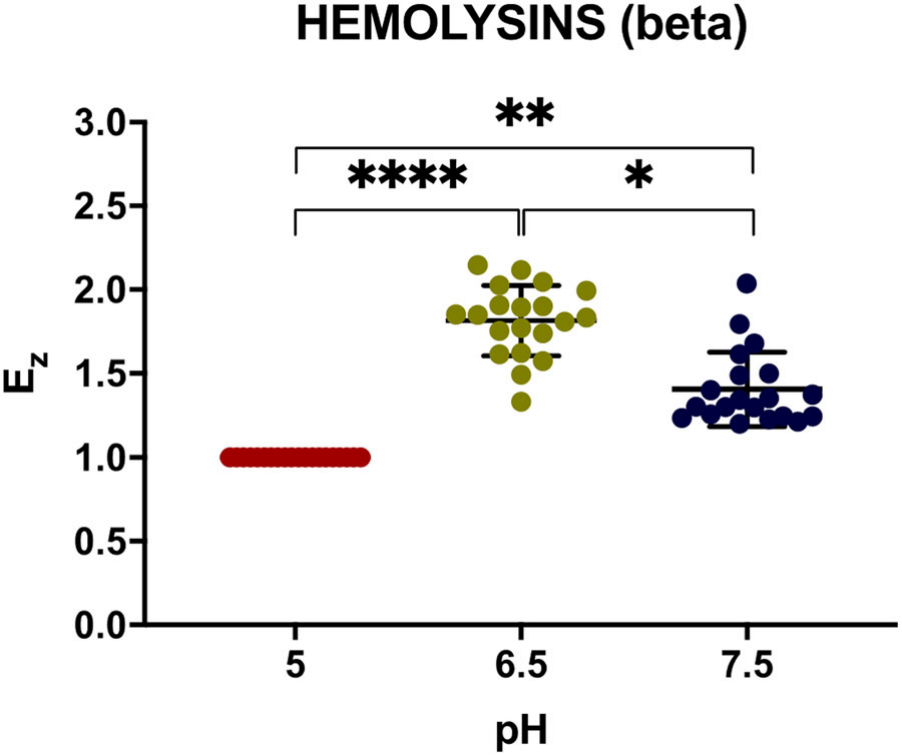
Dependence of the β‐hemolytic activity coefficient (*Ez*) of *Candida albicans* isolates on the tested pH. Scattered red (pH 5), green (pH 6.5), and blue (pH 7.5) circles represent the studied strains. Horizontal bars represent the statistically reliable (*ɑ* = 0.05) mean (longer bar) and standard deviation (shorter bars) values. Brackets indicate statistically comparable sets of data and thresholds (*p ≤ 0.05,**p ≤ 0.01, and ****p ≤ 0.0001).

**Figure 9:**
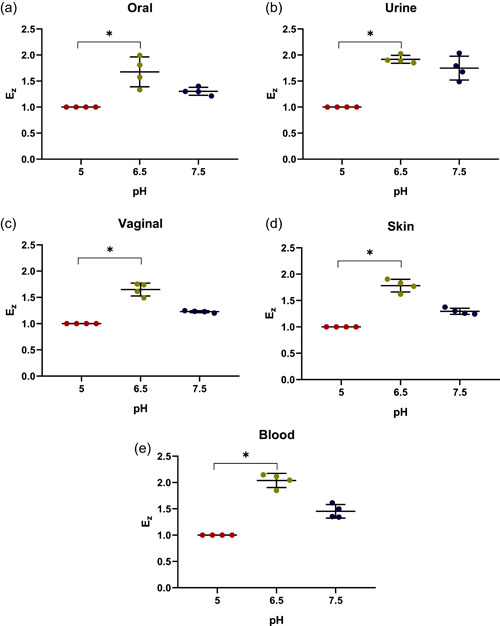
Dependence of the β‐hemolytic activity coefficient (*Ez*) of *Candida albicans* isolates on pH. Assays with *Candida albicans* strains isolated oral cavity (a), urine (b), vagina (c), skin (d), and blood (e) were carried out on blood agar plates at pH 5, 6.5, and 7.5. Scattered red (pH 5), green (pH 6.5), and blue (pH 7.5) circles correspond to the studied strains. Horizontal bars represent the statistically reliable (*ɑ* = 0.05) mean (longer bar) and standard deviation (shorter bars) values. Brackets indicate statistically distinct sets of data (*p ≤ 0.05).

The conditions employed to detect the hemolysin activities of the control strains were next used to assay clinical isolates. A representative set of data obtained with oral cavity isolates is shown in Figure 7 **,** whereas the remaining data are further summarized in Table 4. As seen in this table, the assays carried out with blood agar plates provided detection of β‐hemolysin activities. The assays performed at pH 6.5 and 7.5 revealed the presence of β‐hemolytic activity in all strains, although it was less prominent at pH 7.5 (Figures 7b,c). The subsequent comparison of β‐hemolytic activities determined for all *C. albicans* clinical isolates at all three pHs revealed statistically significant (*p* < 0.0001) differences in the mean value of *Ez* (Figure 8). Moreover, further analysis of these data applying the corrected Dunn's test revealed a dependence of these mean values on the origin of these strains (Figure 9). Furthermore, analysis of β‐hemolysin activities disclosed statistically significant differences between those of vaginal and blood isolates (*p* = 0.02) at pH 6.5 as well as between those of urine and vaginal isolates (*p* = 0.006) at pH 7.5.

**Table 4:** Detection of β‐hemolytic activities of clinical *Candida albicans* isolates from blood, oral cavity, skin, urine, and vagina

	Substrate	Tween 80				Substrate	Tween 80		
pH		5.0	6.5	7.5	pH		5.0	6.5	7.5
	Blood					Oral cavity			
Isolate ID	18‐012	‐	+	+	Isolate ID	08‐052	‐	+	+
	18‐020	‐	+	+		18‐038	‐	+	+
	18‐028	‐	+	+		19‐010	‐	+	+
	19‐002	‐	+	+		19‐078	‐	+	+
	Skin					Urine			
Isolate ID	05‐126	‐	+	+	Isolate ID	07‐154	‐	+	+
	15‐159	‐	+	+		13‐008	‐	+	+
	16‐136	‐	+	+		13‐028	‐	+	+
	93‐432	‐	+	+		15‐153	‐	+	+
	Vagina								
Isolate ID	18‐137	‐	+	+					
	18‐100	‐	+	+					
	18‐105	‐	+	+					
	18‐135	‐	+	+					

*Note*: The presence and absence of detectable β‐hemolysin activities are indicated by ‘+’ and ‘‐’, respectively.

## 4 DISCUSSION

Although *Candida* spp. are conditionally present in the human microbiota as commensal microorganisms, they can also cause human diseases ranging from mild mucosal infections to severe deep‐seated ones. The recent spread of candidiasis is thought to be linked to an increase in the percentage of invasive medical interventions, the emergence of multi‐drug resistant strains, and the growing number of immunocompromised individuals (Ciurea et al., 2020). To exert their pathogenic properties, *Candida* spp. have to produce a number of virulence factors including secreted hydrolytic enzymes that help pathogens to surpass the host defense and develop diseases. Although *C. albicans* can colonize host tissues with very different pH, the impact of this environmental factor on the production and activity of hydrolytic enzymes is largely unknown. Therefore, this study aimed to determine the effect of pH on the activity of hydrolases secreted by *C. albicans* clinical isolates. To achieve this objective, we analyzed the activity of proteases, lipases, and hemolysins secreted by *C. albicans* isolates from five different infection sites (i.e., blood, oral cavity, skin, urine, and vagina) possessing different physiological pH.

While analyzing the impact of pH on secreted proteases, we could detect protease activities only at acidic pH (i.e., at pH 5 and 6.5). These findings suggest that the secreted proteases are likely more active at low pH, such as those found in the human vagina and skin, and therefore might belong to the family of secreted aspartic proteases previously identified in *Candida* (Aoki et al., 2011). Although BSA is more frequently used for the detection of secreted proteases, we showed that the use of both substrates (i.e., BSA and skim milk) made it possible to detect protease activity in a higher number of isolates. Moreover, our data suggest that the use of skim milk instead of BSA (Table 2) could help to reveal more protease‐positive isolates at pH 6.5. Interestingly, our plate assays disclosed that some skin isolates could develop a filamentous phenotype at pH 7.5, which is close to the pH of the blood and internal tissues where mycelial and hyphal forms of some *Candida* spp*.* are found (Yue et al., 2018). However, any association of the above morphological states with cutaneous candidiasis is doubtful because the skin pH is acidic (i.e., 4.7–5.7; Ogawa et al., 1992).

Regarding the urine *C. albicans* isolates, 75% and 50% of them showed protease activity in BSA‐ and skim milk‐containing media, respectively. These results are in concordance with those previously obtained by Alenzi (2016).

Unlike isolates from other sites of infection, oral and blood isolates were 100% protease‐positive on BSA plates at pH 5. These results are consistent with the high percentage (42%–70%) of protease‐positive oral (Hernández‐Solís et al., 2014) and blood (Vieira de Melo et al., 2019) isolates analyzed in other studies. Moreover, in the latter case (Vieira de Melo et al., 2019), 77% of strains manifested protease activities at pH 3, thus suggesting that they are likely conferred by aspartic proteases that are normally more active at acid pH.

Another interesting observation was the lack of protease activity at pH 7.5, which closely mimics the physiological pH in the oral cavity (pH 7.5) and blood (pH 7.4) of healthy individuals (Table 2). This finding suggests that tissue acidification can potentially stimulate protease activities, thereby facilitating *C. albicans* infections.

Thus, despite the differences in physiological pH in each infection site, no significant correlation was found between the origin of the isolate and the corresponding pattern of protease secretion. However, the use of different substrates has revealed some differences in substrate preference. Thus, although *C. albicans* isolates from the vagina and skin were more efficient in degrading skim milk, those isolated from the oral cavity and urinary tract showed better results with BSA, whereas blood isolates were nearly equally efficient in producing hydrolysis zones with both substrates at acid pH.

Besides assaying protease activity, the same *C. albicans* isolates were tested for the presence of secreted lipases using Slifkin's opacity test (Slifkin, 2000). Although several authors have used this method to observe the activity of lipase‐like enzymes (Nouraei et al., 2021; Pandey et al., 2018; Tefiani et al., 2020), the previous assays were primarily performed using the standard media with a pH close to 6.8. Here we tested a wider range of pH including the physiological pH reported for different infection sites (Table 3).

Analysis of all *C. albicans* isolates revealed that 90% of them showed lipolytic activities at pH 5. However, the percentage of lipase‐positive isolates was lower at pH 6.5 (70%) and 7.5 (35%). Tefiani et al. (2020) tested esterase production at pH 5 and 7, obtaining in both cases a higher percentage (85.7%) of isolates efficiently secreting lipase‐like enzymes. In addition, we found that the percentage of lipase‐positive oral isolates was 100% at pH 6.5, which was consistent with the results obtained in other studies at pH 6.8 (Fatahinia et al.,^2015^; Nouraei et al., 2021). In contrast, the high percentage (i.e., 100%) of skin isolates with lipolytic activity detected in our assays at pH 6.5 was notably higher than that (20%) obtained by Skóra et al. (2010) at nearly the same pH (i.e., pH 6.8) using an API ZYM test. These differences suggest that the direct detection of lipase activities on agar plates (this study) can provide higher rates of detection. As to our results obtained at pH 6.5 with blood (75%), vaginal (50%), and urine (25%) isolates, they were consistent with the high occurrence of lipase‐producing strains among *C. albicans* strains associated with blood (56.2%; Pandey et al., 2018) and differed from those (86.2%) obtained by Zafar et al. (2020) for urine isolates. Thus, our data indicate that pH is an important factor in determining the production of lipase‐like enzymes by *C. albicans*. Moreover, the statistically significant differences (*p* < 0.0001) between the means obtained for lipase‐like activities at three different pH, suggest an indirect correlation between pH and lipolytic activity, as higher pH levels resulted in lower enzymatic activities. Moreover, further analysis revealed higher activities of lipase‐like enzymes in oral and skin isolates compared to those from vaginal, blood, and urine ones and this difference looked more pronounced at higher pH, although more isolates need to be analyzed to obtain statistically supported data.

Interestingly, it seems likely that the slightly acidic pH of the human skin (Ogawa et al., 1992) might promote the de‐repression of genes encoding lipases. Their secretion could potentially promote acidification of the site of infection (i.e., due to lipase‐dependent hydrolysis of lipids leading to accumulation of free fatty acids) and therefore might create a microenvironment optimal for other enzymes, such as secreted aspartic proteases, whose action could, in turn, facilitate tissue damage (Hube et al., 2000).

Regarding the oral cavity, the constant mechanical action of saliva creates a true challenge for oral pathogens as it hinders their adhesion to oral tissues. It seems likely that, similar to other pathogenic fungi (Göttlich et al., 1995), *C. albicans* lipases can increase cell adhesion capacity, probably by enhancing the hydrophobicity of *Candida* cells following the lipase‐dependent release of fatty acids. This scenario appears to take place in patients with diabetes mellitus, whose reduced salivary flow, lower pH, and elevated levels of glucose make the oral cavity the perfect microenvironment to allow the transition from commensal to pathogenic yeast (Rodrigues et al., 2019).

In general, we noticed that the isolates with the highest lipolytic activities at pH 5 also possessed it at pH 6.5 and 7.5, whereas isolates with low activity at pH 5 did not exhibit any significant lipolytic activity at higher pH (i.e., pH 6.5 and 7.5) as well. This observation does not necessarily mean that the corresponding isolates lack expression of lipase genes. Instead, it may indicate that the growth conditions might not be optimal for lipase production or detection. Within the *LIP* gene family (*LIP1‐10*), it has been observed that the expression of each gene varies and depends on the site of infection or a particular stage of the disease (Schofield et al., 2005). For example, *LIP1*, *3,* and *9* are expressed more during gastric infections than in the process of oral candidiasis, whereas LIP2 seems to be preferentially produced and functions during gastrointestinal rather than oral infection. Unlike the above genes, which are induced under particular environmental conditions, *LIP4‐8* genes are constitutively expressed (Schofield et al., 2005).

In addition to proteases and lipases, hemolysins represent another important group of secreted virulence factors occasionally used by *Candida* to strive in the host tissues. The results obtained here demonstrate that, despite the lack of any detectable β‐hemolysin activity at pH 5, it was observed for all *C. albicans* isolates at pH 6.5 and 7.5, yielding stronger signal (larger β‐hemolytic zones) at pH 6.5. These findings are consistent with the results obtained by Riceto et al. (2015) who found that 96% of their *C. albicans* isolates were hemolysin producers. Further comparison of *Candida* isolates at pH 6.5 revealed that blood isolates showed the highest β‐hemolytic activities among all isolates tested, and the differences between blood and vaginal isolates were statistically significant. Moreover, an increase in pH to 7.5 reduced the β‐hemolytic activities of all isolates, demonstrating the highest activities for urine isolates (Figure 9b), whereas the lowest one was observed for vaginal isolates. Although β‐hemolysins activities of *C. albicans*, which were likewise isolated from the genitourinary tract, were observed previously (Udayalaxmi & D'Souza, 2014), the corresponding enzymatic indexes were not determined and therefore cannot be compared with those reported here.

## 5 CONCLUSIONS

In conclusion, our data suggest that the activity of hydrolytic enzymes, in particular proteases, lipases, and hemolysins, is generally dependent on pH and is potentially associated with the clinical origin of isolates. On one hand, these data indicate that the differential susceptibility of the host tissues to *Candida* diseases is likely controlled by the pH‐dependent activity of these enzymes. Moreover, *C. albicans* has a regulatory mechanism that affects the pH‐dependent changes in gene expression (Davis, 2003) and it is controlled by the transcription factor Rim101. Furthermore, the importance of this factor in the pH‐dependent regulation of the gene coding for the aspartic protease Sap5 facilitating mucosa invasion by *C. albicans* has already been demonstrated (Villar et al., 2007).

On the other hand, the above findings suggest that due to an increase in the incidence of invasive candidiasis in susceptible patients, emerging antifungal‐resistant strains, and the demand for diagnostic methods, there is a need for a thorough investigation of virulence factors, which could be promising targets for novel antifungal drugs potentially preventing colonization and ulterior disease development. In this context, it would be interesting to carry out a more comprehensive analysis of selected isolates in the future by employing molecular tools to define the nature of the corresponding enzymes and/or toxins in charge of the hydrolytic activities as well as their conservation among clinical isolates.

## AUTHOR CONTRIBUTIONS

**Asier Ramos‐Pardo:** Data curation (equal); formal analysis (equal); investigation (equal); methodology (equal); validation (equal); visualization (supporting); writing – original draft (equal); writing – review and editing (equal). **Rocío Castro‐Álvarez:** Data curation (equal); formal analysis (equal); investigation (equal); methodology (equal); validation (equal); writing original draft (equal); writing – review and editing (equal). **Guillermo Quindós:** Conceptualization (supporting); funding acquisition (lead); investigation (supporting); project administration (supporting); writing – original draft (supporting); writing – review and editing (supporting). **Elena Eraso:** Funding acquisition (equal); investigation (supporting); project administration (supporting); writing – original draft (supporting); writing – review and editing (supporting). **Elena Sevillano:** conceptualization (supporting); formal analysis (equal); funding acquisition (equal); investigation (supporting); methodology (supporting), project administration (equal); Resources (equal); supervision (equal); writing – original draft (equal; writing– review and editing (equal). **Vladimir Kaberdin:** Conceptualization (lead); formal analysis (supporting); funding acquisition (supporting); methodology (supporting); project administration (equal); supervision (lead); writing – original draft (equal); writing – review and editing (equal).

## CONFLICT OF INTEREST

The authors declare that they have no conflicts of interest in the current study. Outside the current study, we declare the following potential conflicts: Guillermo Quindós has received research grants from Astellas Pharma, Pfizer, Merck Sharp & Dohme, and Scynexis. Guillermo Quindós has served on advisory/consultant boards for Merck, Sharp & Dohme, and Scynexis, and he has received speaker honoraria from Abbvie, Astellas Pharma, Merck Sharp & Dohme, Pfizer, and Scynexis.

## ETHICS STATEMENT

None required.

## Data Availability

All data are provided in full in this paper.
